# Daily Intake of Milk Powder and Risk of Celiac Disease in Early Childhood: A Nested Case-Control Study

**DOI:** 10.3390/nu10050550

**Published:** 2018-04-28

**Authors:** Elin M. Hård af Segerstad, Hye-Seung Lee, Carin Andrén Aronsson, Jimin Yang, Ulla Uusitalo, Ingegerd Sjöholm, Marilyn Rayner, Kalle Kurppa, Suvi M. Virtanen, Jill M. Norris, Daniel Agardh, on behalf of the TEDDY Study Group

**Affiliations:** 1 The Diabetes and Celiac Disease Unit, Department of Clinical Sciences, Lund University, 202 05 Malmö, Sweden; elin.malmberg_hard_af_segerstad@med.lu.se (E.M.H.A.S.); carin.andren_aronsson@med.lu.se (C.A.A.); 2 Health Informatics Institute, Morsani College of Medicine, University of South Florida, 33620 FL Tampa, USA; hye-seung.lee@epi.usf.edu (H.-S.L.); jimin.yang@epi.usf.edu (J.Y.); ulla.uusitalo@epi.usf.edu (U.U.); 3 Department of Food Technology, Engineering and Nutrition, Chemical Center, Lund University, 221 00 Lund, Sweden; ingegerd.sjoholm@food.lth.se (I.S.); marilyn.rayner@food.lth.se (M.R.); 4 Tampere Center for Child Health Research, University of Tampere and Tampere University Hospital, 33521 Tampere, Finland; kalle.kurppa@uta.fi; 5 Unit of Nutrition, National Institute for Health and Welfare, 00271 Helsinki, Finland; Faculty of Social Sciences, University of Tampere, Tampere Center for Child Health Research, University of Tampere and Tampere University Hospital and the Science Center of Pirkanmaa Hospital District Tampere, 33521 Tampere, Finland; suvi.virtanen@thi.fi; 6 Department of Epidemiology, Colorado School of Public Health, University of Colorado Anschutz Medical Campus, 80045 CO Aurora, USA; jill.norris@ucdenver.edu

**Keywords:** infant feeding, Sweden, HLA, milk powder, formula, gluten, commercial infant foods

## Abstract

Milk powder and gluten are common components in Swedish infants’ diets. Whereas large intakes of gluten early in life increases the risk of celiac disease in genetically at-risk Swedish children, no study has yet evaluated if intake of milk powder by 2 years of age is associated with celiac disease. A 1-to-3 nested case-control study, comprised of 207 celiac disease children and 621 controls matched for sex, birth year, and HLA genotype, was performed on a birth cohort of HLA-DR3-DQ2 and/or DR4-DQ8-positive children. Subjects were screened annually for celiac disease using tissue transglutaminase autoantibodies (tTGA). Three-day food records estimated the mean intake of milk powder at ages 6 months, 9 months, 12 months, 18 months, and 24 months. Conditional logistic regression calculated odds ratios (OR) at last intake prior to seroconversion of tTGA positivity, and for each time-point respectively and adjusted for having a first-degree relative with celiac disease and gluten intake. Intake of milk powder prior to seroconversion of tTGA positivity was not associated with celiac disease (OR = 1.00; 95% CI = 0.99, 1.03; *p* = 0.763). In conclusion, intake of milk powder in early childhood is not associated with celiac disease in genetically susceptible children.

## 1. Introduction

Celiac disease is a common chronic small bowel disease caused by intolerance to gluten found in foods containing wheat, rye or barley [1]. It has been debated whether the global differences in prevalence are due to variations in infant feeding practices [2]. One affecting factor could be variations in gluten intake during the first years of life [3]. The effects of dairy product intake on the risk of celiac disease is less studied. Although the vast majority of patients with celiac disease have antibodies directed against tissue transglutaminase (tTGA) [4], a proportion also have detectable antibodies against milk protein [5]. Although a recent study did not find avoidance of cow´s milk-based products to protect from celiac disease compared with extensively hydrolyzed formula [5], it is not entirely clear whether other components in milk products may trigger celiac disease.

Commercial instant porridges and cereal milk drinks based on milk powder and gluten containing cereals are common infant food products in some parts of the world [6]. In milk powder production, advanced glycation end products (AGEs) are formed through Maillard reactions [7]. AGEs have pro-inflammatory effects and may induce increased oxidative stress in adults [8]. Notably, levels of AGEs increase during storage of commercial instant porridge and cereal milk drinks in room temperatures [9]. It could therefore be hypothesized that a high intake of commercial instant porridge and cereal milk drinks containing high concentrations of AGEs cause an initial inflammation that results in an increased gut permeability to gluten antigens that eventually leads to celiac disease in genetic at risk individuals.

The aim of this study was to investigate if intake of milk powder is associated with celiac disease in children. We prospectively collected food data from a birth cohort of genetically predisposed children that later developed celiac disease and compared it to matched controls in a nested case-control study.

## 2. Subjects and Methods

### 2.1. Study Population

The Environmental Determinants of Diabetes in the Young (TEDDY) study is an observational study conducted at 6 clinical centers in Finland, Germany, Sweden and the United States, investigating the environmental factors associated with type 1 diabetes and celiac disease [10]. Children carrying any of the HLA genotypes associated with type 1 diabetes and celiac disease were invited to participate in a 15-year follow-up [10], and among the enrolled participants 2525 were from the Swedish site. The TEDDY study is monitored by the National Institutes of Health and has been approved by ethics review boards at individual sites and informed consent from a parent or primary caretaker were obtained prior to screening.

### 2.2. Screening for Celiac Disease

Annual screening for celiac disease begins at 2 years of age by measurement of IgA and IgG autoantibodies against tTGA using radioligand binding assays as previously described [1]. Children positive for tTGA have their blood samples analyzed to determine the closest time point of tTGA seroconversion. Children positive for tTGA in two consecutive samples were evaluated for celiac disease at their health care provider. Diagnosis of celiac disease was established if a child had a biopsy showing Marsh score of 2 or higher and responded to a gluten-free diet with a significant decrease in tTGA levels.

**Figure 1:**
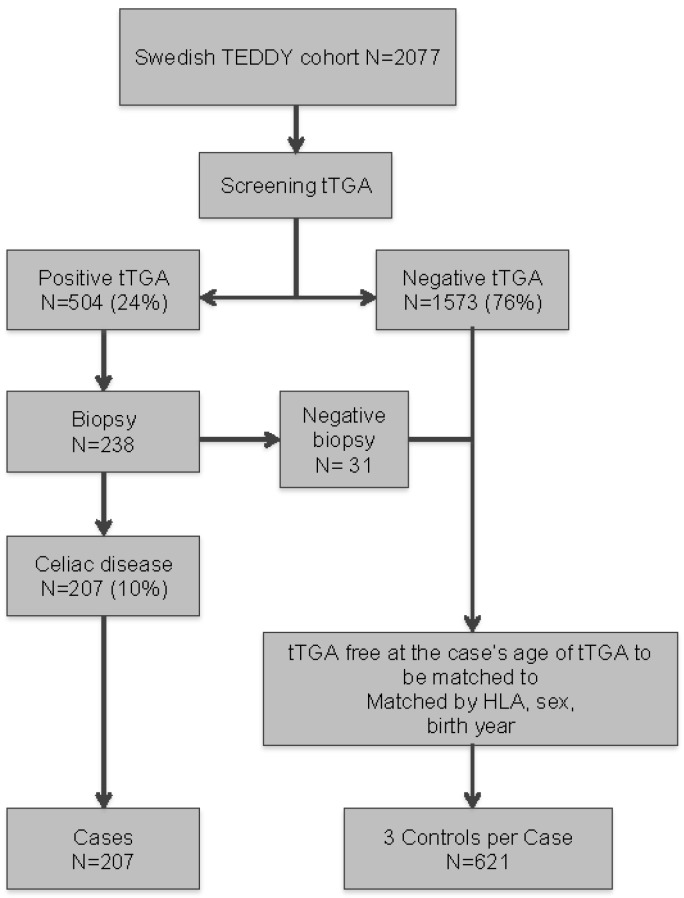
Flowchart of the study population.

As of July 31 in 2016, 2,077 Swedish TEDDY children had been screened for tTGA of whom 504 (24%) were tTGA positive at median 30 months of age (first quartile (Q1): 21, third quartile (Q3): 53) and 85 of those (17%) children seroconverted to tTGA prior to or at 24 months of age. Among the 238 tTGA positive children that were finally investigated with an intestinal biopsy, 207 of the 2077 (10%) children were diagnosed with celiac disease at median 45 months of age (Q1: 33, Q3: 70) (Figure 1).

### 2.3. Study Design

A nested case-control design included the 207 cases with biopsy-proven celiac disease and 3 controls randomly selected from the cohort for each case after matching on gender, the number of HLA DQ2 alleles and birth year (i.e., 1–3 nested case-control design) (Table 1). All controls were free of biopsy-proven celiac disease within 3 months of the matched case’s age of biopsy, as well as tTGA negative within 3 months of the matched case’s age of seroconversion. In this nested case-control study, 39 cases were selected as controls until seroconversion of tTGA.

### 2.4. Dietary Assessment

A study nurse collected a 24-h dietary recall at the first visit between 3 and 4.5 months of age. Three-day food records, including two weekdays and one weekend day, were then collected at follow-up visits at 6 months, 9 months, 12 months, 18 months, and 24 months of age, respectively. Normal food habits were encouraged during the time of the food record collection. Parents were provided with a manual including written instructions, as well as photos of portion sizes and drawings of foods of different sizes and as reference. When the child started daycare, a set of separate food record sheets and manual were provided for the daycare personnel. At the study visits, the study nurse performed a face-to-face interview, probing for missing or unclear information and revising the food record accordingly. A trained study dietitian or nutritionist entered the dietary information in a food database. The TEDDY database for Sweden was based on the Swedish National Food Composition Database, with information about nutrient content for foods and standard recipes for several composite dishes [11]. Products and brands different from standard food items in ingredients or nutritional values as well as unique recipes recorded by families were added to the database. For commercial baby foods, recipes were created based on the ingredient list together with information on the nutritional value, and added as a new food item if it changed in nutritional value or content. The study personnel entering the food data reached consensus estimates for the weight of foods when there was no information in the national food database or from the producer.

Intake of milk powder was either obtained directly from the database (including infant cereal milk drink and instant porridge), as an estimate for average content in a food type (including infant formula and chocolate) or an estimate based on brand name (including yoghurts). Based on the structure of the database, the content of milk powder could not be estimated for some specific products (such as ice cream, powder-based sauces, and certain prepared foods). From the dietary records, gluten intake was also assessed as it was considered a confounding factor. Total intake of wheat, rye and barley could be obtained from the database, and the amount of ingested gluten was calculated by multiplying the analyzed content of protein in each of these grains with 0.8 for wheat, 0.65 for rye, and 0.5 for barley [12].

**Figure 2:**
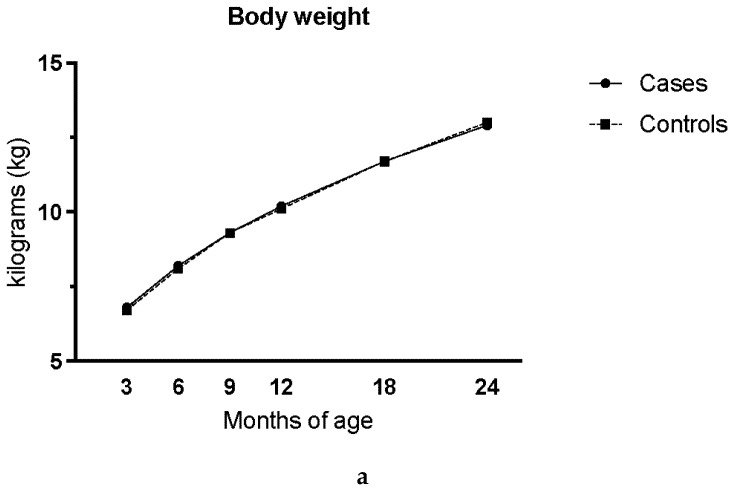

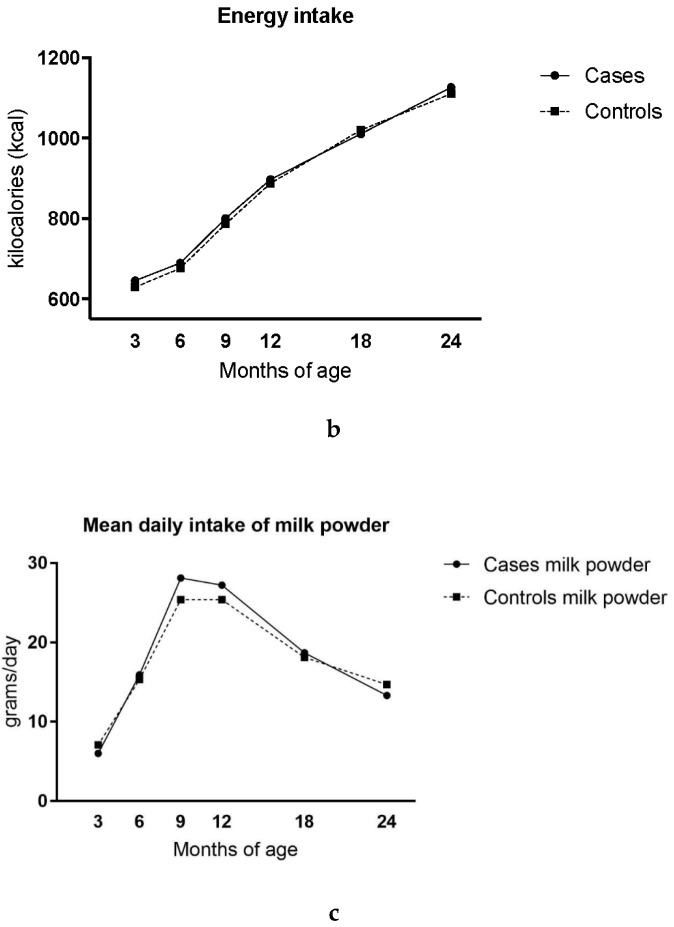
Body weight at clinic visit (**a**) and assessed daily mean intake of energy (kcal) (**b**), mean daily intake of milk powder (gram per day) from 3-day food records in cases with celiac disease and matched, healthy controls (1:3) (**c**). For breastfed subjects, energy intake was estimated using assessed energy requirement and subtracting energy intake from other reported food. Controls were matched to cases by gender, HLA genotype and birth year.

Body weight was measured at every clinic visit at 3 months, 6 months, 9 months, 12 months, 18 months, and 24 months (Figure 2a). Scales were of different brands over the study period of which Tanita (Tanita Corp, Tokyo, Japan) was most commonly applied. Tanita scale was the most common and that scales were calibrated regularly. Energy intake for breastfed subjects was estimated using the energy requirement based on the child’s age and weight at the time for the food record, then subtracting the energy intake from other reported food (Figure 2b) [13].

### 2.5. Statistical Analysis

Daily intake of milk powder in grams per day was assessed as the mean intake from the three-day diet records. Intake data were available until 24 months of age, but age of tTGA seroconversion for cases ranged from 10 to 120 months. Only available intakes reported prior to the case’s tTGA seroconversion were included in analysis in order to compare between a case and matched control, using conditional logistic regression. Daily intake was also analyzed in grams per kilogram body weight (g/kg/day) in order to standardize intake between subjects. Intake reported at the visit prior to the case’s tTGA seroconversion was as “last intake,” and the sum of all intakes assessed as “total intake.” Intake at a given age (3 months, 6 months, 9 months, 12 months, 18 months, or 24 months) was compared if it was available or appropriate for the case’s age of seroconversion. We first adjusted for having a first-degree relative with celiac disease as a confounder. Milk powder intake was then analyzed with and without adjustment for gluten intake in order to separate the effect on risk of celiac disease between the two dietary exposures. Odds ratios were reported with 95% confidence intervals (CI), along with two-sided p-value. Statistical significance was determined when *p*-value <0.05. All statistical analyses were performed using SAS version 9.4 (SAS Institute Inc., Cary, NC, USA).

## 3. Results

Energy intake and body weight increased as expected with age for both cases and controls (Figure 2a,b). The mean daily intake of milk powder increased in both cases and controls by 12 months of age and more significantly between 6 months and 9 months of age, respectively (Figure 2c). At 6 months, the intake of milk powder was 15.9 g and 15.3 g per day for cases and controls respectively. At 9 months it had increased to 28.1 grams and 25.4 grams per day for cases and controls and at 24 months it had decreased to 13.3 grams and 14.7 grams per day for cases and controls.

Neither energy intake nor body weight were associated with celiac disease. Intake of milk powder in grams per day prior to seroconversion of tTGA positivity did not increase the risk of celiac disease, either for last intake, nor total intake or for intake at any given age. This was also true for the relative intake in grams per kg body weight (Table 2). In the unadjusted model, there was a small increased risk for celiac disease for the milk powder intake at 9 months of age in grams per day (OR = 1.01, 95%CI = 1.0–1.02; *p* = 0.037), as well as in grams per kilogram bodyweight per day (OR = 1.1, 95%CI = 1.0–1.2; *p* = 0.044).

Having a first-degree relative with celiac disease (OR = 2.53, 95%CI 1.37, 4.67, *p* = 0.003) and reported gluten intake when assessed in grams per day (OR = 1.09, 95%CI 1.03–1.16; *p* = 0.004) as well as in grams per kilograms per day (OR = 2.73, 95%CI 1.36–5.49; *p* = 0.005) were associated with celiac disease. When these confounders were included the adjusted model, the association between milk powder intake and celiac disease no longer remained significant.

## 4. Discussion

The present study showed that intake of milk powder does not increase the risk of celiac disease in genetically susceptible Swedish children. The peak intake of milk powder was observed at the age of 9 months after which it started to decrease, and may reflect a dietary intake pattern of formula, commercial porridge and milk cereal drink. The intake of milk powder observed was equivalent to the amount of commercial porridge and cereal milk drink consumed by Swedish infants as reported in a previous study [6].

The strength of the dietary assessment methods used in this study is that they allow for estimations of individual intake of foods. Repeated food records measure changes in dietary habits of infants and growing young children over time and is a suitable method when studying dietary intake and risk of disease [14]. Another advantage with the dietary data collected for this study is minimization of recall bias, which has been a limitation in previous studies using retrospective dietary assessment methods [15,16]. A prospective study design has the advantage of unawareness of the tTGA status at the time of the food data collection, which otherwise may influence parents to change their child’s diet.

Analysis of relative dietary intake we also made, as recommended in nutrition research and disease [17]. The energy requirement of a child depends on age, weight, and growth [13]. A child larger in size may consume bigger portions than their smaller counterpart, resulting in a higher nutrient intake, but not necessarily higher intake in relation to body weight. We adjusted for the confounders of having a family member with celiac disease and for the gluten intake, respectively. The adjustments had a significant impact on the results, since we found an association with increased risk for celiac disease and intake of milk powder reported at the 9-month visit. We have previously published a study on the association between amount of gluten intake and celiac disease performed on the same cohort, which showed that the last intake of every gram gluten per day before seroconversion to tTGA positivity increased the risk of celiac disease by 28% [3].

The limitations of this study includes that milk powder intake was only studied in the first 2 years of life, whereas the majority of the celiac cases were diagnosed several years later. As 15.6% of the subjects also had missing food record data at 24 months, data collected at earlier timepoints was applied for the analyses, which may affect the reliability of the results. Additionally, we did not have access to complete information on content of milk powder for all food items; therefore, estimates had to be used. Although the excluded foods were considered less commonly used in the selected age groups, estimations on possible amounts of missing data were not performed. In this study, we analyzed two dietary exposures, and some common Swedish infant products contain both. Although the statistical analyses adjusted for the gluten intake, the study could be criticized for not quantifying the amount of gluten from products that also contained milk powder.

In conclusion, this nested case-control study on intake of milk powder during the first 2 years of life in genetically susceptible children showed that consumption of milk powder is not associated with celiac disease for Swedish children.

## Figures and Tables

**Table 1:** Characteristics of the identified cases with celiac disease in the Swedish TEDDY birth cohort used as matching factors in a nested 1-3 case-control study.

Matching Variable	Cases N = 207 (%)
Sex	
- Female	131 (63.3)
- Male	76 (36.7)
Birth year	
- 2004	11 (5.3)
- 2005	39 (18.8)
- 2006	28 (13.5)
- 2007	41 (19.8)
- 2008	37 (17.9)
- 2009	46 (22.2)
- 2010	5 (2.4)
HLA-genotype	
- DQ2/DQ8	64 (30.9)
- DQ8/DQ8	35 (16.9)
- DQ2/DQ2	100 (48.3)
- Other	8 (3.9)

**Table 2:** Comparison of mean daily intake of milk powder (grams per day and grams per kilo bodyweight) in cases with celiac disease and matched, healthy controls (1:3), and risk of celiac disease analyzed with conditional logistic regression expressed as odds ratio (OR) after adjusting for having a first-degree relative with celiac disease and for gluten intake at the given time point. Controls were matched to cases by gender, HLA genotype and birth year.

Time point	Number of cases analyzed	Number of cases missing intake	Milk powder intake (g/day)		Milk powder intake (g/kg/day)	
			OR (CI 95%)	*p*-value	OR (CI 95%)	*p*-value
Last intake ^1^	207	0	1.0 (0.99–1.01)	0.937	0.99 (0.87–1.13)	0.861
Total intake ^2^	207	0	1.0 (1.0–1.00)	0.662	1.0 (0.98–1.03)	0.763
Intake at						
3 months	207	0	0.99 (0.97–1.01)	0.159	0.92 (0.83–1.02)	0.125
6 months	202	5	1.0 (0.99–1.02)	0.643	1.02 (0.91–1.13)	0.788
9 months	198	9	1.01 (1.0–1.02)	0.069	1.09 (0.99–1.19)	0.072
12 months	192	9	1.01 (1.0–1.02)	0.181	1.08 (0.96–1.21)	0.184
18 months	146	21	1.0 (0.98–1.02)	0.983	1.01 (0.84–1.21)	0.923
24 months	103	19	0.99 (0.97–1.01)	0.202	0.88 (0.69–1.12)	0.301

^1^ Last reported intake at the visit prior to seroconversion of tTGA; ^2^ Sum of all reported intakes prior to seroconversion of tTGA

## Colorado Clinical Center

Marian Rewers, M.D., Ph.D., PI^1,4,5,6,10,11^, Kimberly Bautista^12^, Judith Baxter^9,10,12,15^, Ruth Bedoy^2^, Daniel Felipe-Morales, Kimberly Driscoll, Ph.D.^9^, Brigitte I. Frohnert, M.D.^2,14^, Marisa Gallant, M.D.^13^, Patricia Gesualdo^2,6,12,14,15^, Michelle Hoffman^12,13,14^, Rachel Karban^12^, Edwin Liu, M.D.^13^, Jill Norris, Ph.D.^2,3,12^, Adela Samper-Imaz, Andrea Steck, M.D.^3,14^, Kathleen Waugh^6,7,12,15^, Hali Wright^12^. University of Colorado, Anschutz Medical Campus, Barbara Davis Center for Childhood Diabetes.

## Finland Clinical Center

Jorma Toppari, M.D., Ph.D., PI^￥^1,4,11,14^, Olli G. Simell, M.D., Ph.D.^￥^1,4,11,13^, Annika Adamsson, Ph.D.^^12^, Suvi Ahonen^*±§^, Heikki Hyöty, M.D., Ph.D.^*±6^, Jorma Ilonen, M.D., Ph.D.^￥¶3^, Sanna Jokipuu^^^, Tiina Kallio^^^, Leena Karlsson^^^, Miia Kähönenμ^¤^, Mikael Knip, M.D., Ph.D.^*±5^, Lea Kovanen^*±§^, Mirva Koreasalo^*±§2^, Kalle Kurppa, M.D., Ph.D.^*±13^, Tiina Latva-ahoμ^¤^, Maria Lönnrot, M.D., Ph.D.^*±6^, Elina Mäntymäki^^^, Katja Multasuoμ^¤^, Juha Mykkänen, Ph.D.^￥ 3^, Tiina Niininen±^*12^, Sari Niinistö^±§2^, Mia Nyblom^*±^, Petra Rajala^^^, Jenna Rautanen^±§^, Anne Riikonen^*±§^, Mika Riikonen^^^, Minna Romo^^^, Juulia Rönkäμ^¤^, Jenni Rouhiainen^^^, Tuula Simell, Ph.D., Ville Simell^^￥13^, Maija Sjöberg^￥^12,14^, Aino Steniusμ^¤12^, Maria Leppänen^^^, Sini Vainionpää^^^, Eeva Varjonen^￥^12^, Riitta Veijola, M.D., Ph.D.^μ¤14^, Suvi M. Virtanen, M.D., Ph.D.^*±§2^, Mari Vähä-Mäkilä^^^, Mari Åkerlund^*±§^, Katri Lindfors, Ph.D.^*13￥^ University of Turku, ^*^University of Tampere, μUniversity of Oulu, ^^^Turku University Hospital, Hospital District of Southwest Finland, ±Tampere University Hospital, ^¤^Oulu University Hospital, ^§^National Institute for Health and Welfare, Finland, ^¶^University of Kuopio.

## Georgia/Florida Clinical Center

Jin-Xiong She, Ph.D., PI^1,3,4,11^, Desmond Schatz, M.D.^*4,5,7,8^, Diane Hopkins^12^, Leigh Steed^12,13,14,1^5, Jamie Thomas^*6,12^, Janey Adams^*12^, Katherine Silvis^2^, Michael Haller, M.D.^*14^, Melissa Gardiner, Richard McIndoe, Ph.D., Ashok Sharma, Joshua Williams, Gabriela Young, Stephen W. Anderson, M.D.^^^, Laura Jacobsen, M.D.^*14^ Center for Biotechnology and Genomic Medicine, Augusta University. ^*^University of Florida, ^^^Pediatric Endocrine Associates, Atlanta.

## Germany Clinical Center

Anette G. Ziegler, M.D., PI^1,3,4,11^, Andreas Beyerlein, Ph.D.^2^, Ezio Bonifacio Ph.D.^*5^, Michael Hummel, M.D.^13^, Sandra Hummel, Ph.D.^2^, Kristina Foterek^￥2^, Nicole Janz, Mathilde Kersting, Ph.D.^￥2^, Annette Knopff^7^, Sibylle Koletzko, M.D.^¶13^, Claudia Peplow^12^, Roswith Roth, Ph.D.^9^, Marlon Scholz, Joanna Stock^9,12,14^, Katharina Warncke, M.D.^14^, Lorena Wendel, Christiane Winkler, Ph.D.^2,12,15^. Forschergruppe Diabetes e.V. and Institute of Diabetes Research, Helmholtz Zentrum München, and Klinikum rechts der Isar, Technische Universität München. ^*^Center for Regenerative Therapies, TU Dresden, ^¶^Dr. von Hauner Children’s Hospital, Department of Gastroenterology, Ludwig Maximillians University Munich, ^￥^Research Institute for Child Nutrition, Dortmund.

## Sweden Clinical Center

Åke Lernmark, Ph.D., PI^1,3,4,5,6,8,10,11,15^, Daniel Agardh, M.D., Ph.D.^13^, Carin Andrén Aronsson^2,12,13^, Maria Ask, Jenny Bremer, Ulla-Marie Carlsson, Corrado Cilio, Ph.D., M.D.^5^, Emelie Ericson-Hallström, Lina Fransson, Thomas Gard, Joanna Gerardsson, Rasmus Bennet, Monica Hansen, Gertie Hansson, Susanne Hyberg, Fredrik Johansen, Berglind Jonsdottir, M.D., Helena Elding Larsson, M.D., Ph.D. ^6,14^, Marielle Lindström, Markus Lundgren, M.D.^14^, Maria Månsson Martinez, Maria Markan, Jessica Melin^12^, Zeliha Mestan, Karin Ottosson, Kobra Rahmati, Anita Ramelius, Falastin Salami, Sara Sibthorpe, Birgitta Sjöberg, Ulrica Swartling, Ph.D.^9,12^, Evelyn Tekum Amboh, Carina Törn, Ph.D. ^3,15^, Anne Wallin, Åsa Wimar^12,14^, Sofie Åberg. Lund University.

## Washington Clinical Center

William A. Hagopian, M.D., Ph.D., PI^1,3,4, 5, 6,7,11,13, 14^, Michael Killian^6,7,12,13^, Claire Cowen Crouch^12,14,15^, Jennifer Skidmore^2^, Josephine Carson, Maria Dalzell, Kayleen Dunson, Rachel Hervey, Corbin Johnson, Rachel Lyons, Arlene Meyer, Denise Mulenga, Alexander Tarr, Morgan Uland, John Willis. Pacific Northwest Diabetes Research Institute.

## Pennsylvania Satellite Center

Dorothy Becker, M.D., Margaret Franciscus, MaryEllen Dalmagro-Elias Smith^2^, Ashi Daftary, M.D., Mary Beth Klein, Chrystal Yates. Children’s Hospital of Pittsburgh of UPMC.

## Data Coordinating Center

Jeffrey P. Krischer, Ph.D.,PI^1,4,5,10,11^, Michael Abbondondolo, Sarah Austin-Gonzalez, Maryouri Avendano, Sandra Baethke, Rasheedah Brown^12,15^, Brant Burkhardt, Ph.D.^5,6^, Martha Butterworth^2^, Joanna Clasen, David Cuthbertson, Christopher Eberhard, Steven Fiske^9^, Dena Garcia, Jennifer Garmeson, Veena Gowda, Kathleen Heyman, Francisco Perez Laras, Hye-Seung Lee, Ph.D.^1,2,13,15^, Shu Liu, Xiang Liu, Ph.D.^2,3,9,14^, Kristian Lynch, Ph.D. ^5,6,9,15^, Jamie Malloy, Cristina McCarthy^12,15^, Steven Meulemans, Hemang Parikh, Ph.D.^3^, Chris Shaffer, Laura Smith, Ph.D.^9,12^, Susan Smith^12,15^, Noah Sulman, Ph.D., Roy Tamura, Ph.D.^1,2,13^, Ulla Uusitalo, Ph.D.^2,15^, Kendra Vehik, Ph.D.^4,5,6,14,15^, Ponni Vijayakandipan, Keith Wood, Jimin Yang, Ph.D., R.D.^2,15^. Past staff: Lori Ballard, David Hadley, Ph.D., Wendy McLeod. University of South Florida.

## Project scientist

Beena Akolkar, Ph.D.^1,3,4,5,6,7,10,11^. National Institutes of Diabetes and Digestive and Kidney Diseases.

## Autoantibody Reference Laboratories

Liping Yu, M.D.^^5^, Dongmei Miao, M.D.^^^, Polly Bingley, M.D., FRCP^*5^, Alistair Williams^*^, Kyla Chandler^*^, Saba Rokni^*^, Claire Williams^*^, Rebecca Wyatt^*^, Gifty George^*^, Sian Grace^*^. ^^^Barbara Davis Center for Childhood Diabetes, University of Colorado Denver, ^*^School of Clinical Sciences, University of Bristol UK.

## HLA Reference Laboratory

Henry Erlich, Ph.D.^3^, Steven J. Mack, Ph.D., Anna Lisa Fear. Center for Genetics, Children’s Hospital Oakland Research Institute.

## Repository

Sandra Ke, Niveen Mulholland, Ph.D. NIDDK Biosample Repository at Fisher BioServices.

## Committees

^1^Ancillary Studies, ^2^Diet, ^3^Genetics, ^4^Human Subjects/Publicity/Publications, ^5^Immune Markers, ^6^Infectious Agents, ^7^Laboratory Implementation, ^8^Maternal Studies, ^9^Psychosocial, ^10^Quality Assurance, ^11^Steering, ^12^Study Coordinators, ^13^Celiac Disease, ^14^Clinical Implementation, ^15^Quality Assurance Subcommittee on Data Quality.

## Trial Identification Number

NCT00279318 (www.clinicaltrials.gov).
